# Human Infection with Eurasian Avian-Like Swine Influenza A(H1N1) Virus, the Netherlands, September 2019

**DOI:** 10.3201/eid2703.201863

**Published:** 2021-03

**Authors:** Anna Parys, Elien Vandoorn, Jacqueline King, Annika Graaf, Anne Pohlmann, Martin Beer, Timm Harder, Kristien Van Reeth

**Affiliations:** Ghent University, Merelbeke, Belgium (A. Parys, E. Vandoorn, K. Van Reeth);; Friedrich-Loeffler-Institut, Greifswald Insel-Riems, Germany (J. King, A. Graaf, A. Pohlmann, M. Beer, T. Harder)

**Keywords:** influenza A virus, influenza virus, viruses, H1N1 subtype, avian-like, respiratory infections, swine, humans, zoonoses, the Netherlands

## Abstract

We report a zoonotic infection of a pig farmer in the Netherlands with a Eurasian avian-like swine influenza A(H1N1) virus that was also detected in the farmed pigs. Both viruses were antigenically and genetically characterized. Continued surveillance of swine influenza A viruses is needed for risk assessment in humans and swine.

Eurasian avian-like swine influenza A(H1N1) viruses (IAVs) are entirely derived from a precursor virus of avian origin (*1*) and have been enzootic in the swine population in Europe since 1979 and in Asia since 1993. Zoonotic infections with such viruses, which are then termed H1N1 variant (H1N1v) viruses, occur sporadically. Most cases occur in humans who have direct exposure to pigs. Since 1986, several human cases of Eurasian avian-like H1N1 swine IAV have been reported in Europe (*2**–**4*) and China (*3*,*5*).

These events reflect the possibility of Eurasian avian-like H1N1 swine IAV transmission from swine to humans. In this study, we report an infection with a Eurasian avian-like H1N1 swine IAV in a pig farmer and his pigs in a herd in the Netherlands. We also conducted whole-genome characterization of viruses from the man and the pigs.

## The Study

On September 18, 2019, acute respiratory disease was observed in a 43-year-old man (farmer) and his 14-week-old fattening pigs and gilts. The pigs of this closed farm showed coughing, anorexia, tachypnea, dyspnea, and lethargy. Two days earlier, a 44-year-old man (animal caretaker) had reported similar symptoms. The sows of this herd (n = 420) were vaccinated against swine IAVs with Respiporc FLU3 vaccine (Ceva, https://www.ceva.com), but the farmer and animal caretaker were not recently vaccinated against human seasonal influenza viruses. Both humans and the pigs recovered completely within 10 days after the first appearance of signs or symptoms. Family members and close contacts of the men did not show development of influenza-like symptoms.

Six days after onset of disease, nasal swab samples were collected from the farmer, the animal caretaker, and 6 symptomatic pigs. Human samples were collected by self-sampling, and informed consent was obtained from the farmer and the animal caretaker. Subsequently, samples were shipped to the Laboratory of Virology, Faculty of Veterinary Medicine, Ghent University (Merelbeke, Belgium).

Upon inoculation into MDCK cells, IAV was isolated from the sample of the farmer and from a pooled sample of the pigs; no virus was isolated from the animal caretaker. Public health authorities in the Netherlands were notified about the H1N1v infection. The human H1N1v isolate was named A/Netherlands/Gent-193/2019, and the swine H1N1 isolate was named A/swine/Netherlands/Gent-193/2019.

Virus neutralization tests with swine antiserum against swine IAVs of the H1N1, H1N2, and H3N2 subtypes showed an antigenic relationship between both newly discovered isolates and Eurasian avian-like H1N1 swine IAVs from 1998 and 2010, as well as the prototype influenza A(H1N1)pdm09 (pH1N1) A/California/04/2009 virus. Serologic cross-reactivity with H1N2 or H3N2 swine IAVs was not observed (Table 1).

**Table 1:** Cross-reactivity in virus neutralization tests between isolates from a pig farmer and his pigs and reference swine H1N1, pH1N1, H1N2 and H3N2 viruses*

Virus	Subtype	H1 clade	Virus neutralization titer for swine antiserum†						
			swBe98	swG10	Ca09	swG99	swG12	swFl98	swG08
swBe98	H1N1	1C.2	**4,096**	256	96	32	<4	<4	<4
swG10	H1N1	1C.2.1	48	**768**	12	<4	<4	<4	<4
Ca09	pH1N1	1A.3.3.2	12	96	**1,536**	<4	<4	<4	<4
swG99	H1N2	1B.1.2.1	6	<4	4	**1,024**	768	<4	<4
swG12	H1N2	1B.1.2.1	<4	<4	6	768	**1,536**	<4	<4
swFl98	H3N2	NA	<4	<4	4	<4	<4	**8,129**	512
swG08	H3N2	NA	<4	<4	<4	<4	<4	3,072	**768**
Ne19	H1N1v	1C.2.2	128	64	8	<4	<4	<4	<4
swNe19	H1N1	1C.2.2	256	384	1,536	8	<4	<4	<4

*Ne19 indicates isolate from the farmer; swNe19 indicates the isolate from the pigs. Homologous titers are indicated in bold. H1N1v, H1N1 variant; NA, not applicable; pH1N1, influenza A(H1N1)pdm09; swBe98, A/swine/Belgium/1/98; swG10, A/swine/Gent/28/2010; Ca09, A/California/04/2009; swG99, A/swine/Gent/7625/99; swG12, A/swine/Gent/26/2012; swFl98, A/swine/Flanders/1/98; swG08, A/swine/Gent/172/2008; Ne19, A/Netherlands/Gent-193/2019; swNe19, A/swine/Netherlands/Gent-193/2019. †Swine antiserum was obtained by a double vaccination with whole inactivated virus vaccines.

Initial analyses by multiplex real-time reverse transcription PCRs (*6*) and whole-genome next-generation sequencing (*7*) of both isolates confirmed that all genome segments were closely related to those of Eurasian avian-like H1N1 swine IAVs. A BLAST homology search (http://www.fludb.org) with both whole genomes showed highest nucleotide identities (96%) for hemagglutinin (HA) and neuraminidase with clade 1C.2.2 Eurasian avian-like H1N1 swine IAVs isolated in Germany and the Netherlands during 2011–2012. These databases contain limited numbers of sequences of this swine IAV clade, which explains the lack of similar recent viruses. A phylogenetic tree of Eurasian avian-like H1N1 swine IAVs isolated in Europe and Asia was constructed by using MEGA7 software (https://www.megasoftware.net). Phylogenetic analysis confirmed the genetic relationship of the HA1 genes of both isolates with Eurasian avian-like H1N1 swine IAVs of clade 1C.2.2 (Table 2; Figure).

**Table 2:** Influenza virus sequences downloaded from GenBank, GISAID, or unpublished data and used in phylogenetic analysis*

Isolate	Country	Collection date	Date of download	Accession no.
A/swine/Finistere/2899/82	France	1982	2019 Nov 16	AJ344015
A/Netherlands/386/86	Netherlands	1986	2019 Nov 16	AF320065
A/Netherlands/477/93	Netherlands	1993	2019 Nov 16	AF320066
A/swine/Denmark/19126/93	Denmark	1993	2019 Nov 16	KC900289
A/swine/Netherlands/609/96	Netherlands	1996	2019 Nov 16	AF320064
A/swine/Belgium/1/98	Belgium	1998	2019 Nov 17	AY590824
A/swine/Italy/1513–1/98	Italy	1998	2019 Nov 16	CY116458
A/Switzerland/8808/2002	Switzerland	2002	2019 Nov 16	AJ517815
A/swine/Spain/50047/2003	Spain	2003	2019 Nov 7	CY009892
A/swine/Spain/53207/2004	Spain	2004	2019 Nov 17	KR700597
A/swine/Zhejiang/1/2007	China	2007 Nov 15	2019 Nov 7	FJ415610
A/swine/Germany/SIV04/2008	Germany	2008 Jun	2019 Nov 16	FN429078
A/swine/France/CotesdArmor-0388/2009	France	2009 Jul 28	2019 Nov 17	KC881265
A/swine/Gent/28/2010	Belgium	2010 Jan 13	2019 Jul 29	KP406525
A/Jiangsu/1/2011	China	2011 Jan 4	2019 Nov 23	KF057112
A/swine/Jiangsu/40/2011	China	2011 Jan 9	2019 Nov 23	JQ319648
A/swine/Germany/Wunnenberg-IDT13220/2011	Germany	2011 Mar 31	2019 Dec 16	KR699726
A/swine/Germany/Reinberg-IDT14457–1/2012	Germany	2012 Jan 2	2019 Dec 16	KR700366
A/swine/Netherlands/Dalfsen-12/2012	Netherlands	2012 Jan 10	2019 Dec 16	KR700020
A/swine/Germany/Ellerbrock-IDT14696/2012	Germany	2012 Jan 18	2019 Dec 16	KR700389
A/swine/Gent/62/2015	Netherlands	2015 Mar 19	NA	Unpub. data†
A/Hunan/42443/2015	China	2015 Jul 2	2020 Jun 15	EPI206573‡
A/swine/Gent/173/2015	Belgium	2015 Sep 4	NA	Unpub.data†
A/Pavia/65/2016	Italy	2016 Oct	2020 Mar 27	KY368150
A/Netherlands/3315/2016	Netherlands	2016 Oct	2020 Mar 27	KY250319
A/swine/Gent/150/2016	Belgium	2016 Nov 18	NA	Unpub. data†
A/swine/Gent/196/2018	Belgium	2018 Oct 12	NA	Unpub. data†
A/swine/Gent/241/2018	Belgium	2018 Nov 4	NA	Unpub. data†
A/swine/Gent/05/2019	Belgium	2019 Jan 9	NA	Unpub. data†
A/swine/Gent/54/2019	Belgium	2019 Mar 13	NA	Unpub. data†
A/swine/Netherlands/Gent-193/2019	Netherlands	2019 Sep 24	2020 Apr 29	MT395373
A/Netherlands/Gent-193/2019	Netherlands	2019 Sep 24	2020 Apr 29	MT395365
A/swine/Gent/203/2019	Belgium	2019 Oct 9	NA	Unpub. data†

*Accession numbers are from GenBank HYPERLINK  except as indicated. NA, not applicable. †Ghent University (Merelbeke, Belgium). ‡GISAID, https://www.gisaid.org.

**Figure:**
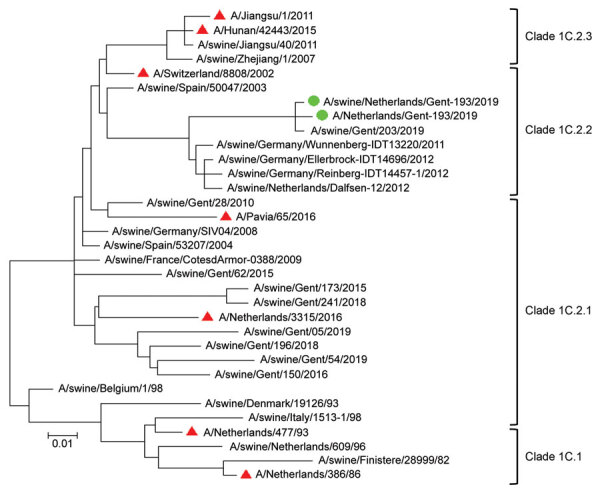
Phylogenetic tree based on amino acid sequences of the hemagglutinin 1 of Eurasian avian-like swine influenza A(H1N1) virus isolates from a pig farmer and his pigs (green circles), the Netherlands, and reference sequences (see Table 2). Red triangles indicate reference sequences from humans. Phylogenetic relationships were estimated by using the maximum-likelihood method in MEGA7 software (https://www.megasoftware.net) and the Jones-Taylor-Thornton substitution model with a gamma distribution of among-site rate. Branch length is proportional to genetic distance. Scale bar indicates amino acid substitutions per site.

The human H1N1v and swine H1N1 isolates differed in several positions in the genes coding for the 3 polymerase proteins (polymerase basic [PB] 2, PB1, and polymerase acidic), the HA gene, and the nonstructural protein gene. The other 3 gene segments (neuraminidase, nucleoprotein, and matrix) were 100% identical. HA gene sequences of the swine H1N1 and the human H1N1v isolates showed amino acid substitutions K142N, N195S, and V215I (H1 numbering). Position 142 is located in antigenic site Ca2 and position 195 in antigenic site Sb (*8*). In human seasonal influenza A(H1N1) viruses, a substitution at position 142 was reported to cause antigenic change (*9*). This substitution might explain the loss of reactivity with pH1N1 antiserum for the human H1N1v isolate versus the swine H1N1 isolate (Table 1). In addition, in H5 IAVs this substitution decreased the pH at which the HA underwent fusion (*10*).

Because human-adapted viruses undergo fusion at a lower pH (5.0–5.5) than swine-adapted and avian-adapted viruses (pH 5.6–6.0), such mutations might contribute to human adaptation of zoonotic viruses. We found multiple substitutions in the polymerase genes of the human H1N1v isolate: R739Q in PB2, L108I and T652A in PB1, and D682N in polymerase acidic. Based on analyses in the FluSurver database (https://flusurver.bii.a-star.edu.sg/), the R739Q substitution in the PB2 gene might influence the binding of PB2 to host protein(s). We also found 2 substitutions in the nonstructural protein gene: V18I and G227R. Sequences were made publicly available in GenBank (accession nos. MT395362–77), and GISAID (https://www.gisaid.org; accession nos. EPI_ISL_430866 [A/swine/Netherlands/Gent-193/2019] and EPI_ISL_0865 [A/Netherlands/Gent-193/2019]).

## Conclusions

We report another zoonotic infection with a Eurasian avian-like H1N1 swine IAV in Europe since the emergence of the virus in 1979 (*2**–**4*). No further human-to-human transmission was reported, although it cannot be excluded that the farmer was infected by the animal caretaker. The nasal swab sample from the caretaker might have tested negative because it was collected as late as 8 days after he reported influenza-like symptoms.

The swine antiserum against the pH1N1 virus cross-reacted with the swine H1N1 isolate from this investigation (Table 1) but had a 192-fold lower virus neutralization titer against the human H1N1v isolate. Therefore, it is unlikely that current human seasonal vaccines would provide cross-protection against the human H1N1v isolate. This finding is consistent with our recent investigations of human serum samples for antibodies against 8 H1 swine IAVs representing 7 predominant H1 clades of swine IAVs; only 55 (10%) of 549 human serum samples had hemagglutination inhibition titers >40 against a European avian-like H1N1 swine IAV of clade 1C.2.1, which is predominant in swine in Europe (*11*), compared with 24%–54% against 5 other clades (*12*). These data point toward a relatively greater zoonotic risk for avian-like H1N1 swine IAVs from Europe and are consistent with previous studies about Eurasian avian-like H1N1 swine IAVs from China (*5**,**13*). Our data further support the notion that Eurasian avian-like H1N1 swine IAVs need to be monitored closely.

We found several amino acid substitutions between the H1N1 swine isolate and the H1N1v human isolate, but their role remains obscure. The past 2 decades have seen an unprecedented increase of data for putative mammalian-adaptive mutations of avian influenza viruses. The known genetic markers are mainly based on studies with wholly avian viruses of various HA subtypes in mammalian cell culture or in ferrets. Knowledge of amino acid substitutions that might enable adaptation of swine-adapted influenza viruses to humans, in contrast, is almost nonexistent (*14*). This finding is true for Eurasian avian-like H1N1 swine IAVs, as well as for the pH1N1 virus, which is the only known swine-origin virus with the ability to spread efficiently between humans. Our study highlights the need for experimental research on this topic and for continued surveillance of swine IAVs because of the risk for human infection or zoonotic spread.
